# Species traits determined different responses to “zero-growth” policy in China’s marine fisheries

**DOI:** 10.1038/s41598-022-24897-w

**Published:** 2022-11-27

**Authors:** Bin Kang, Linlong Wang, Min Liu

**Affiliations:** 1 Key Laboratory of Mariculture, Ocean University of China, Ministry of Education, Qingdao, Shandong China; 2 Fisheries College, Ocean University of China, Qingdao, Shandong China; 3 State Key Laboratory of Marine Environmental Science, College of Ocean and Earth Sciences, Xiamen University, Xiamen, Fujian China

**Keywords:** Population dynamics, Ichthyology, Ocean sciences

## Abstract

China remains the largest nation of marine capture fisheries in the world in the last few decades, at the cost of offshore fisheries degradation by overfishing. Although fisheries regulations have become gradually tightened, the recovering evidences are weak and the catch species compositions are far from satisfactory. To explore better and reasonable countermeasures, besides the “zero growth” policy (i.e. the national total fisheries production limitation), five targets with different ecological traits were selected for stock assessment and rebuilding by Monte Carlo Catch-Maximum Sustainable Yield method. The results showed the control of total rather than species catch could not lead to the recovery of fisheries and maintain community function. Individual species showed different responses to overfishing according to their biological characteristics. High trophic level species can be sensitive to overfishing, and difficult to rebuild stocks after collapse. Pelagic small fish resources increased first but eventually decreased under high fishing pressure. Scientific-based restocking can enhance resource recovery. Besides “zero growth” policy, fisheries management should be further refined, in particular for main economic species based on their biological traits, as well as the support of reliable fisheries statistics and regulation implementation in place. To relieve the conflict between rising fishery products demand and falling catches, aquaculture and seeking resources from the high seas and EEZs are supposed to be successful ways, on the premise of taking full account of ecological health, maritime safety, and food security.

## Introduction

Four seas around China, including the Bohai Sea, the Yellow Sea, the East China Sea and the South China Sea, are semi-closed marginal, resulting in relatively independent domestic marine fisheries that lack of worldwide distributed species and pelagic migratory fish stocks^1^. However, China remains the largest nation of marine capture fisheries in the world in the last few decades, with a catch volume of 9.474 million tons (MT) and a value of 219.72 billion CNY in 2020^2^. Such high catches in China’s exclusive economic zones (EEZ) place enormous pressures not only on the sustainability of China indigenous fisheries stocks but also global fish stocks^3,4^.

China has been engaged in marine fishery production statistics since 1950. Benefit from the development of fishing technology and the increase of employees, the total amount of marine catches dramatically increased about 25 times from 0.6 MT in 1950 to 15 MT in 1998, and then maintained the volumes of 10–15 MT for nearly 2 decades^5^. Despite such the apparent increase of capture volumes, various results have shown the significant declines in traditional fisheries stocks and the changes of species compositions in catches. Fishing gears are diverse in China such as trawling, stow net, purse seine, drift gillnet, dip net, square net, squid jigging, long-line fishing, pole fishing, and cage fishing^1^. Among these, trawling contributes the most at 46% of the domestic marine fishery catches, followed by stow net (17%), drift gillnet (14%), and purse seine (9%)^6^. In 2020, China has 136,800 marine fishing vessels registered with a capacity of 13.44 million kW^2^. This poses great challenges to the sustainable utilization and the effective management of China's domestic marine fisheries.

China has been developed a philosophy of fishery management since the 1950s. In June 1955, The State Council issued the “Order Regarding Motorized Trawlers Closed Fishing Areas of the Bohai Sea, the Yellow Sea and the East China Sea”. Since the late-1970s, the fishery management measures in China have shown more diverse. In 1979, the “Regulations on the Breeding and Protection of Aquatic Resources” were promulgated, for the first time proposing a fishing license system as an administrative regulation. In 1986, the national “Fisheries Law” was issued, which marked that China's fishery management entered a new era of “ruling fishing by law”. A series of regulations focusing on EEZ fisheries management, including the “dual control” of fishing vessels on the total number and the total horsepower since 1987 and the “summer fishing moratorium” since 1995. In 1999, the Ministry of Agriculture decided to implement the “zero-growth” policy for controlling the total marine capture volumes, and further strengthened to “negative growth” regulation in 2000. The projected marine capture volumes were required to maintain at a level no higher than Year 1998 (i.e. less than 15 MT), indicating that China has shifted its focus from pursuing rapid growth in fisheries production to sustainable using natural resource. To compensate the employee income loss from fishing production limitation, the “dual transfer” system of fishing vessels was issued in 2002, and “fishing fuel subsidies” was introduced in 2006. In 2017, the “total resources management” and “quota fishing management” were promulgated. The regulation details include the reduction on annual marine capture production quota control and the total number and horsepower of fishing vessels, and the extension of the summer fishing closure period^1,6,7^.

It is difficult to evaluate whether the fishery regulations are success or effective because the catch species composition is far from satisfactory. The traditionally commercial species of high-trophic levels were gradually replaced by small, low-trophic species to support steady domestic marine catch volumes^5^, with about 57% of traditional fish stocks overexploited or collapsed^8^. Therefore, the assessment of fisheries status not only depends on the total production, but also on the species composition and the dominant species. The main catch species were the hairtail *Trichiurus* spp. (mainly *T. japonicus* and *T. lepturus*), the large yellow croaker *Larimichthys crocea*, the small yellow croaker *Larimichthys polyactis*, and the cuttlefishes (mainly as *Sepiella maindroni* and *Sepia esculenta*) in the 1950s and 1960s, approximate 35% of the total domestic marine capture productions. The filefishes *Thamnaconus* spp., the Chub mackerel *Scomber japonicus* and the Japanese scad *Decapterus maruadsi* joined the dominant species group in the 1970s and 1980s, contributing approximate 15% of the total domestic marine capture productions. By the 1990s, the capture production of the butterfish *Pampus argenteous* and the anchovy *Engraulis japonicas* increased rapidly; in the 2000s the dominant species group enrolled a new member, the South American pilchard *Sardinops sagax*, as well as a certain number of crustaceans and cephalopods^2,9^. In 2010, 80% of the marine catch production in China's EEZ came from fully developed (66%) and developed (15%) stocks, and the rest was from overexploited (18%) stocks^10^. It is obvious that the “zero-growth” policy on the total catch production limitation did little to protect single species or local ecosystem function.

There is a lack of in-depth assessment of the effectiveness of Chinese fishery regulations. Except recent publications on assessment of individual commercial species in certain areas^11–13^, studies on the changes of catch species composition, stock and species mortality under overfishing, as well as fisheries management, development strategy and policy assessment are little. The information concerning on marine fisheries released by the Chinese government is almost in Chinese, and so is much of the scientific literature, which causes obstacles for international exchanges and cooperation. The Monte Carlo Catch-Maximum Sustainable Yield method (CMSY) has been applied to estimate fisheries reference points from catch, resilience, and stock status at the start year and the end year on data-limited stocks, with specially emphasis on deriving informative priors for productivity, unexploited stock size, catch ability, and biomass. The biomass and fishing mortality of CMSY evaluation can provide references for resource rebuilding^14,15^. CMSY estimates the general resilience and productivity of the stock instead of the species, which means that species interactions between different stocks and environmental impacts are implicitly considered, suggesting the availability of this method for a multi-species model on a certain area^15^. In recent years, this method has been widely used to assess the status of fishery species in different regions^16,17^.

In this study, CMSY was used to describe the status of China's domestic marine capture fisheries and evaluate the effectiveness of “zero-growth” policy. The fisheries rebuilding under different fishing pressures was also simulated. To further understand the community composition and ecosystem function, five species/species groups with different ecological traits were selected for stock assessment and rebuilding. The piscivorous *Trichiurus* spp. have ranked absolutely the first in China's domestic marine fishery productions since 1956, and suffered great fishing pressure. Another traditional economic species is *L. polyactis*, a species mainly feeding on planktonic crustaceans, which is a link of many organisms in the food chain^5^. Pelagic species *S. sagax*, a by-catch species, can regulate the population of other species through down-up regulation mechanism^18^. In addition, the swimming crab *Portunus trituberculatus* and the cuttlefishes (mainly *S. maindroni* and *S. esculenta*, unless specified) were selected as representatives of crustaceans and cephalopods, respectively. Understanding the response patterns of different ecological species to fishing pressure and “zero-growth” policy will help formulate detailed, feasible and effective management and reach sustainable use of fishery resources.

## Materials and methods

### Catch data source

The catch can be considered as a series of yields from the available biomass at a given productivity. Data of the total catch and selected species were extracted from Chinese Fishery Statistical Yearbooks^2,9^. The catch volumes of *Trichiurus* spp. and *L. polyactis* were first recorded in 1956, of *P. trituberculatus* in 1987, of *S. sagax* in 1989 and the cuttlefishes in 1989 (Supplementary).

### Parameter determination

The maximum intrinsic rate of population increase (*r*), catch data, and stock status at the beginning year (*B/k*_start_) and the end year (*B/k*_end_) are required to determine the fishery reference indices, including unexploited stock size *k*, viable *r-k* pairs and the fishery reference points, e.g., maximum sustainable yield (*MSY*), *B/B*_msy_, *F/F*_msy_, using CMSY approach^15^.

The value *r* was obtained from Fishbase (www.fishbase.org). If a numeric value is given, it can be used directly; if a state description is given, the default range (high, medium, low) for that type is taken, corresponding to respective value from Froese et al*.*^14^ (Table 1). Considering that total fisheries consist of a mixture of all species, medium for default range of *r* was used in the assessment.

**Table 1:** Priori ranges of *r* and *B*/*k* for CMSY.

*r* (intrinsic growth rate)^a,b^		*B*/*k* (relative biomass)^b, c^	
Resilience	Priori *r* range	Depletion status	Priori *B*/*k* range
High	0.6–1.5	Low depletion	0.4–0.8
Medium	0.2–0.8	Medium depletion	0.2–0.6
Low	0.05–0.5	Strong depletion	0.01–0.4
Very low	0.015–0.1	Very strong depletion	0.01–0.2

^a^Referred from Fishbase (www.fishbase.org); ^b^referred from Froese et al*.*^14^; ^c^referred from Froese et al*.*^19^.

CMSY requires "expert" prior information of biomass consumption specified at the beginning and the end of the time series. In 1951, due to the small number of fishing vessels and the low exploitation rate, *B*/*k*_start_ was defined as low depletion; in 2019, *B*/*k*_end_ was defined as strong depletion because of acknowledged resource destroy (Table 2).

**Table 2:** The prior range for *r* and *B*/*k* and other information of the selected species or species groups in China.

Species/species group	Time series	Resilience	Prior*r*range	*B*/*k* at start year	*B*/*k* at end year
Total	1951–2019	Medium	0.2–0.8	0.4–0.8	0.01–0.4^a^
*Trichiurus *spp.	1956–2019	Medium	0.53–1.20^b^	0.4–0.8	0.01–0.4^c^
*Larimichthys polyactis*	1956–2019	Medium	0.37–0.85^b^	0.01–0.4^d^	0.2–0.6^c^
*Sardinops sagax*	1989–2019	Medium	0.36–0.82^b^	0.4–0.8	0.01–0.4^e^
*Portunus trituberculatus*	1987–2019	High	0.79–1.79^b^	0.4–0.8	0.2–0.6^e^
Cuttlefishes	1989–2019	Medium	0.2–0.8	0.4–0.8	0.01–0.4^e^

^a^Referred from Zhang et al*.*^20^; ^b^referred from Fishbase (www.fishbase.org); ^c^referred from Zhai and Pauly^21^; ^d^referred from Zhuang^22^; ^e^referred from Martell and Froese^23^.

The priori range of *k* (carrying capacity) was determined by Eq. (1) for lower relative biomass or Eq. (2) with higher relative biomass in the end year^14^. CMSY would subsequently draw the most probable *r*–*k* combination (geometric mean) to calculate *B*_t+1_, *MSY*, *B*_msy_, *F*_t_, and *F*_msy_ using Eqs. (3–7)^24,25^ (Table 3).

**Table 3:** Equations for parameters determination in CMSY.

Equations	Code
*k*_low_ = $\max (C)/r_{{{\text{high}}}}$ and *k*_high_ = $4\max (C)/ - r_{{{\text{low}}}}$	(1)
*k*_low_ = $2\max (C)/r_{{{\text{high}}}}$ and *k*_high_ = $12\max (C)/r_{{{\text{low}}}}$	(2)
*B*_t+1_ = *B*_t_ + *r* (1 − $B_{t} /k$) *B*_t_—*C*_t_	(3)
*MSY* = *r* × *k*/4	(4)
*B*_msy_ = *k*/2	(5)
*F*_t_ = ${{C}}_{{{t}}} /{B}_{{{t}}}$	(6)
*F*_msy_ = − ln (1 − ${{MSY}}/{B}_{{{\text{msy}}}}$) = *r*/2	(7)
$B_{{t + 1}} /B_{{{\text{msy}}}} = B_{t} /B_{{{\text{msy}}}} + 2F_{{{\text{msy}}}} B_{t} /B_{{{\text{msy}}}} (1 - B_{t} /2B_{{{\text{msy}}}} {\text{)}} - B_{t} /B_{{{\text{msy}}}} F_{t}$, if $B_{t} /{B}_{{{\text{msy}}}}$ ≥ 0.5	(8)
$B_{{t + 1}} /B_{{{\text{msy}}}} = B_{t} /B_{{{\text{msy}}}} + 2F_{{{\text{msy}}}} 2F_{{{\text{msy}}}} B_{t} /B_{{{\text{msy}}}} (1 - B_{t} /2B_{{{\text{msy}}}} {\text{)}} - B_{t} /B_{{{\text{msy}}}} F_{t}$, if $B_{t} /B_{{{\text{msy}}}}$ < 0.5	(9)
$$ F_{{{\text{reduced}}}} {{ }} = {{ }}2B/B_{{{\text{msy}}}} {\mkern 1mu} F $$	(10)

*k* the carrying capacity, *k*_low_ and *k*_high_ the lower and upper bounds for *k* respectively, *r* the maximum intrinsic rate, *r*_low_ and *r*_high_ the bounds for priori *r* values, *B*_t+1_ the biomass in year t + 1, *C*_t_ the catch in year t, *max (C*) the recorded maximum catch, *MSY* the maximum sustainable yield, *B*_t_ the biomass in year t, *B*_msy_ the biomass capable of producing best maximum sustainable yields, *F*_t_ the fishing mortality in year t, *F*_msy_ the fishing mortality capable of producing maximum sustainable yields.

A Kobe plot was used to explain the fishery status based on the positions of the time series of fishing pressure (*F*/*F*_msy_) on the Y-axis and of population status (*B*/*B*_msy_) on the X-axis. The first quadrant, upper right, indicated stock was in depletion by overfishing (status: destroying); the second quadrant, upper left, indicated stock size was capable of producing high yields close to MSY under reasonable fishing pressure (status: health), that is, the target for management; the third quadrant, lower left, indicated stock was in gradual recovery benefited from reduced fishing pressure (status: recovering); the fourth quadrant, lower right, indicated stock size was too small to sustain population continuity under ongoing overfishing (status: depletion)^19^.

### Fisheries rebuilding

Fish mortality (*F*) plays a decisive role in the stock recovery, directly determined by the ratio of catch to biomass. According to *F*, as well as *B*_msy_ and *F*_msy_ estimated by the CMSY model, the biomass in the next year (*B*_t+1_) was calculated by Eqs. (8–9) from the Schaefer model^24^ (Table 3).

To evaluate the effects of fishing pressure on fisheries recovery, possibly helping for making feasible and effective regulations, three exploitation scenarios were used to predict the stock rebuilding status until 2030: 0.5 *F*_msy_, no fishing scenario, i.e., when *B* < 0.5 *B*_msy_ or *F* = 0.5 *F*_msy_; 0.8 *F*_msy_, sustainable exploitation scenario, i.e., *F* = 0.8 *F*_msy_ when *B* ≥ 0.5 *B*_msy_, or *F* linearly reduced to 0 along with the decrease in biomass when *B* < 0.5 *B*_msy_; 1.0 *F*_msy_, exploitation scenario, i.e., *F* = 1.0 *F*_msy_ in any cases. The reduction in the fishing mortality (*F*_reduced_) was calculated in Eq. (10)^26^ (Table 3).

### Assessment under “zero-growth” policy

Whatever fisheries management regulations are essentially reducing fishing intensity and fishing mortality. Assuming that the “zero-growth” policy is not implemented, fishing pressure would keep the previous trend from 1999 onwards and increase yearly. Year-to-year trend of fishing mortality were determined based on the *F* data from 1989 to 1998, which was used to calculate *F* for each subsequent year since 1999. The resource states of the total catch and selected species were simulated, and the resources rebuilding were also predicted.

All the analyses were executed in R^27^. CMSY R codes were downloaded from http://oceanrep.geomar.de/34476/ and revised accordingly.

## Results

### Total domestic marine capture fisheries

**Figure 1:**
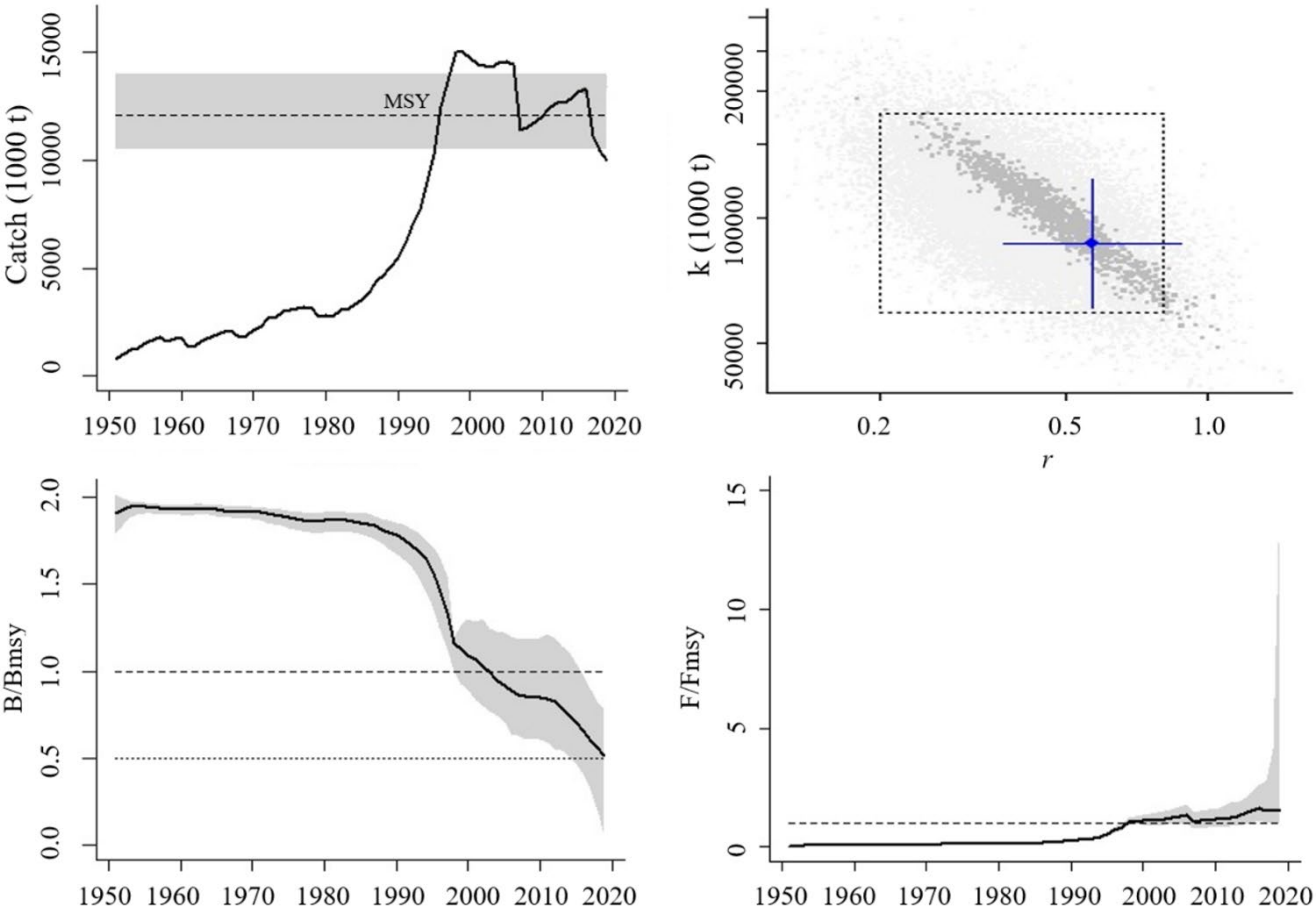
Results of the CMSY. The upper left panel shows catches relating to the *MSY* estimated by CMSY, with indication of 95% confidence limits (CL) in grey. The upper right panel shows the most probable *r–k* pair and its approximate 95% CL in blue. The lower left panel shows the development of relative total biomass (*B*/*B*_msy_), with the grey area indicating uncertainty. The lower right graph shows relative fishing mortality (*F*/*F*_msy_).

Analyses on total domestic marine capture fisheries covered a long-term dataset from the start year 1951 and to the end year 2019 (68 years). The marine capture fisheries production firstly counted and recorded at the start year was 814,800 T. Since the 1980s, the catch increased rapidly and reached the climax at 14.97 MT in 1998. Affected by the “zero-growth” policy, the fisheries output showed a decline trend in the oscillation, down to nearly 10 MT in 2019 (Fig. 1). Altogether 1184 viable trajectories for 1074 *r–k* pairs were found and the final estimates were, viz.: *r* = 0.571, 95% confidence limits (CL) = 0.379–0.861, *k* = 86.59 MT, 95% CL = 61.88–121.17, and *MSY* = 12.09 MT, 95% CL = 10.64–13.96.

**Figure 2:**
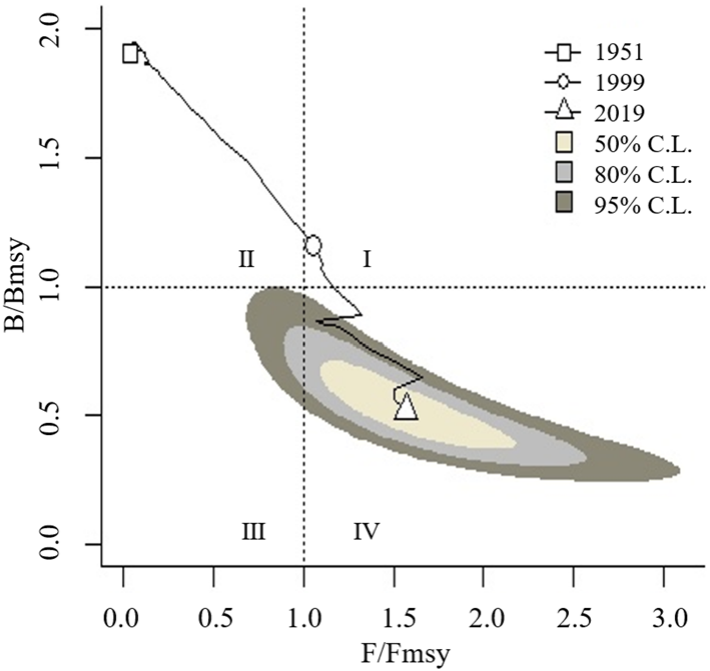
Diagram of fishery status of China marine capture fisheries, based on the time series of fishing pressure (*F/F*_msy_) and of population status (*B*/*B*_msy_), at different confidence limits. 1951 and 2019, the start year and the end year of official statistics; 1999, the first year of the “zero-growth” policy.

The status of China marine capture fisheries showed a declining trend with the time passage, falling into different status in stages (Fig. 2): in 1951–1997, *B*/*B*_msy_ and *F*/*F*_msy_ dropped in the second quadrant, with a linear decline of *B*/*B*_msy_ from 1.95 to 1.16 and a boom of *F*/*F*_msy_ from 0.034 to 0.84; in 1998, *B*/*B*_msy_ was 1.158, and *F*/*F*_msy_ exceeded 1, indicating the resources began to be depleted; after 1998, *F*/*F*_msy_ exceeded 1, indicating the resources remain depleting. Despite the implementation of the “zero-growth” policy, due to the continuous decline of biomass, the value of *B*/*B*_msy_ continued to decline below 1 in 2003 and continuously dropped to 0.52 in 2019. The status of depletion was in the fourth quadrant with *F*/*F*_msy_ 1.56.

**Figure 3:**
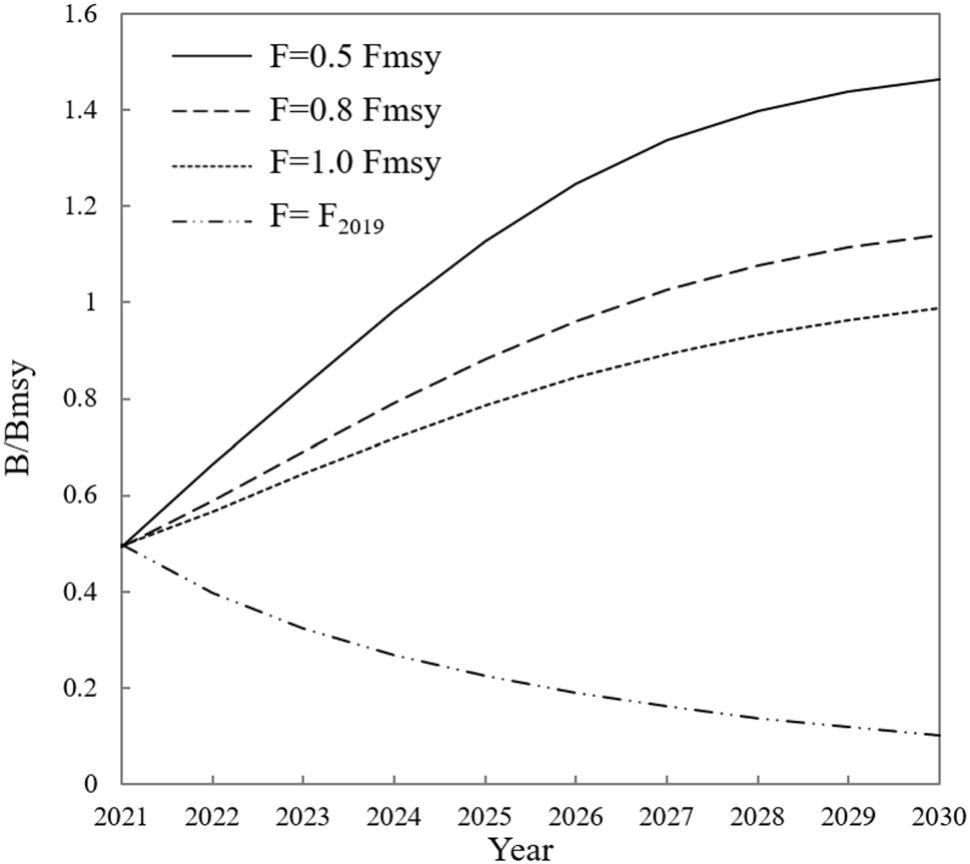
Rebuilding of *B/B*_msy_ in 2021–2030 under different simulating scenarios. The goal of resource management is that the remaining amounts of fisheries should be larger than the volume of biomass capable of producing MSY (*B* > *B*_msy_ or *B* /*B*_msy_ > 1).

Under the rebuilding scenario of *F*/*F*_msy_ = 0.5, the resources reached a healthy state (*B* > *B*_msy_) by 2025 with *B*/*B*_msy_ 1.13, and this value increased to 1.46 by 2030 (Fig. 3). The catch reached 9.1 MT in 2030, which was still a little short of the optimal sustainable utilization point of 12 MT, indicating that 0.5 *F*_msy_ still need to be maintained for a period. Under the 0.8 *F*_msy_ scenario *B*/*B*_msy_ exceeded 1 in 2027, and was 0.99 by 2030 under the 1.0 *F*_msy_ scenario, close to the recovery of national marine fisheries. If the current fishing pressure of 1.56 *F*_msy_ is maintained, *B*/*B*_msy_ will drop to 0.10 by 2030, leaving only 1.6 MT of production. Without any intervention, the current fishing pressure will lead to the depletion of the entire marine fisheries and the degradation of the community function.

**Figure 4:**
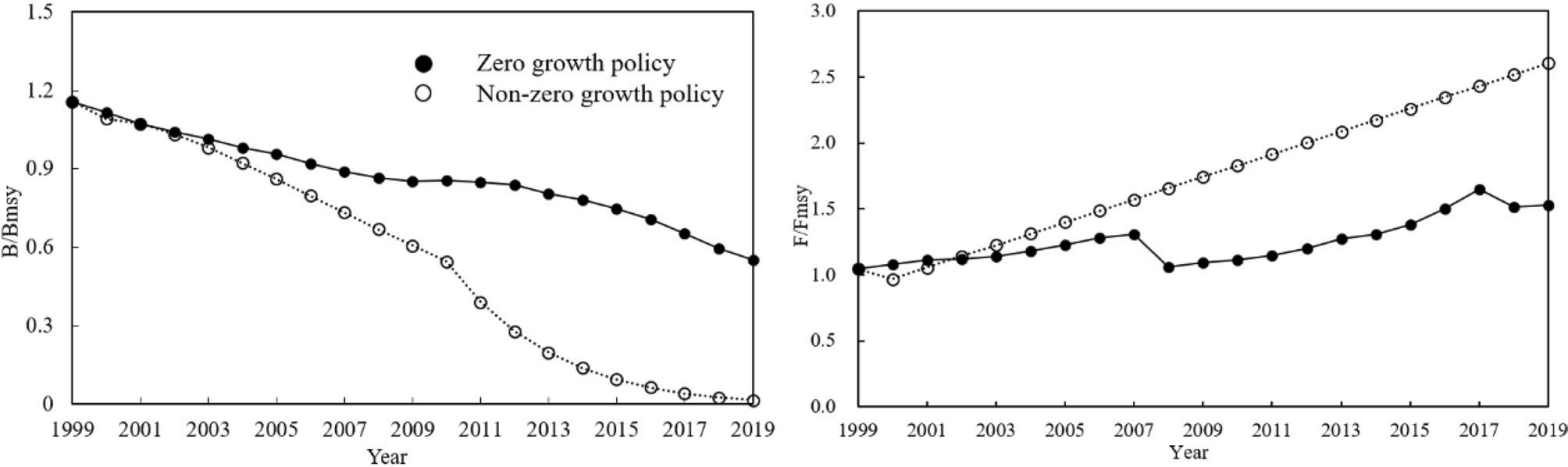
The values of *B*/*B*_msy_ and *F*/*F*_msy_ from 1999 to 2019. Solid and dash lines mean under and without “zero-growth” policy, respectively.

Without the implementation of the “zero-growth” policy in 1999, fishing pressure was assumed to develop in accordance with existing trends, as *F* = 0.024613 year–48.924275 (*R*^2^ = 0.8774), and *F* linearly increased from 0.277 in 1999 to 0.769 in 2019. *B*/*B*_msy_ showed a more obvious downward trend than practical variation; in 2019 *B*/*B*_msy_ decreased to no higher than 0.01 without catch control, only 1% of the value in 1999. Correspondingly, in 1999–2019 *F*/*F*_msy_ increased 2.77 times to 2.690, suggesting the exhaustion of fisheries (Fig. 4). Rebuilding the fisheries under ongoing fishing pressure, biomass increased slightly even if *F* = 0.5 *F*_msy_ was adopted, and there was no possibility of recovery to health.

### Representative species stocks

**Figure 5:**
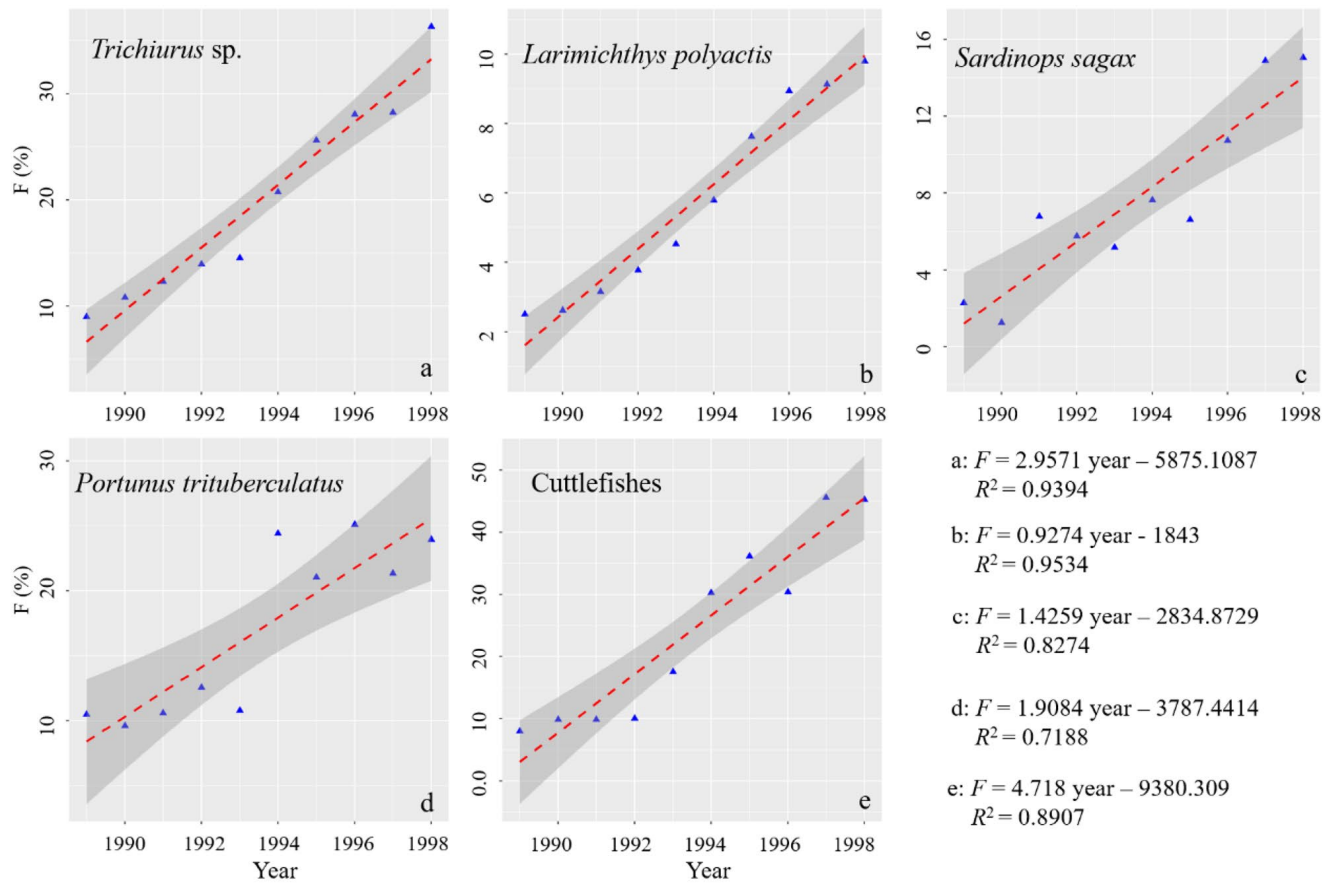
The trend of *F* without “zero-growth” policy of selected species or species group.

The parameters used for assessing the state of selected species, and the estimated biological references were listed in Table 4. The higher the intrinsic growth rate *r* is, the faster the stocks recover. During 1989 and 1998 without the “zero-growth” policy, *F* of selected species showed respective increasing trend (Fig. 5).

**Table 4:** Parameters *r* (intrinsic growth rate), *k* (carrying capacity), *F*_msy_, *B*_msy_, and *MSY* of selected species.

Species	*r*	*k* (T)	*MSY* (T)	*B*_msy_ (T)	*F*_msy_
*Trichiurus* spp.	0.876	519.2	112.3	258.6	0.438
*Larimichthys polyactis*	0.496	279.3	34.6	139.7	0.248
*Sardinops sagax*	0.597	98.6	14.7	49.3	0.298
*Portunus trituberculatus*	1.36	134.7	45.5	67.4	0.681
Cuttlefishes	0.888	78.3	17.3	39.1	0.444

#### *Trichiurus* spp.

**Figure 6:**
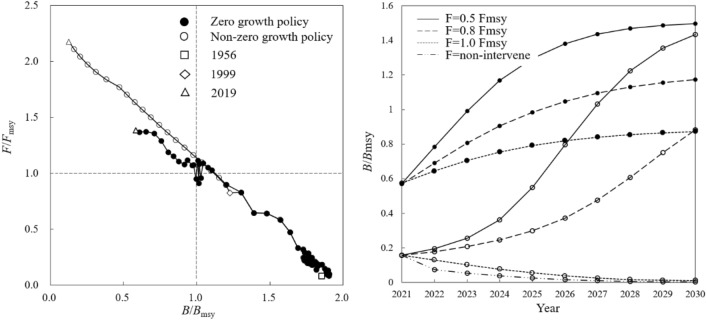
Rebuilding status of *Trichiurus* spp. Resource status in different quadrants following explanation in Fig. 2.

*B*/*B*_msy_ of *Trichiurus* spp. was 1.85 in 1956. Under the continuously growing fishing pressure, *B*/*B*_msy_ declined to below 1 in 2007, and 0.59 in 2019. Accordingly, *F*/*F*_msy_ continuously increased from 0.08 in 1956, breaking 1 for the first time in 2008, to 1.38 in 2019 (Fig. 6, left). Under different scenarios of resource rebuilding, *B*/*B*_msy_ could reach 1.17 in 2024 and 1.50 in 2030 under 0.5 *F*_msy_, or reach 1.04 in 2026 and 1.17 in 2030 under 0.8 *F*_msy_, or 0.87 by 2030 under 1.0 *F*_msy_ (Fig. 6, right).

Without “zero-growth” policy, *B*/*B*_msy_ fell below 1 as early as in 2004 while *F*/*F*_msy_ reached 1.16, indicating a state as “depletion” of resource. The *B*/*B*_msy_ further declined to 0.13 in 2019, only 20% of the practical value (Fig. 6, left). Without rebuilding, *B*/*B*_msy_ would fall close to 0 by 2027 and resources will be exhausted. If rebuilding, under 0.5 *F*_msy_ scenario *B*/*B*_msy_ could recover and reach 1 in 2027 and 1.43 in 2030, with the production of 610,000 T and 830,000 T, respectively. In 0.8 *F*_msy_ scenario, *B*/*B*_msy_ of 2030 was 0.88, while in 1.0 *F*_msy_ scenario there was no recovery (Fig. 6, right).

#### Larimichthys polyactis

**Figure 7:**
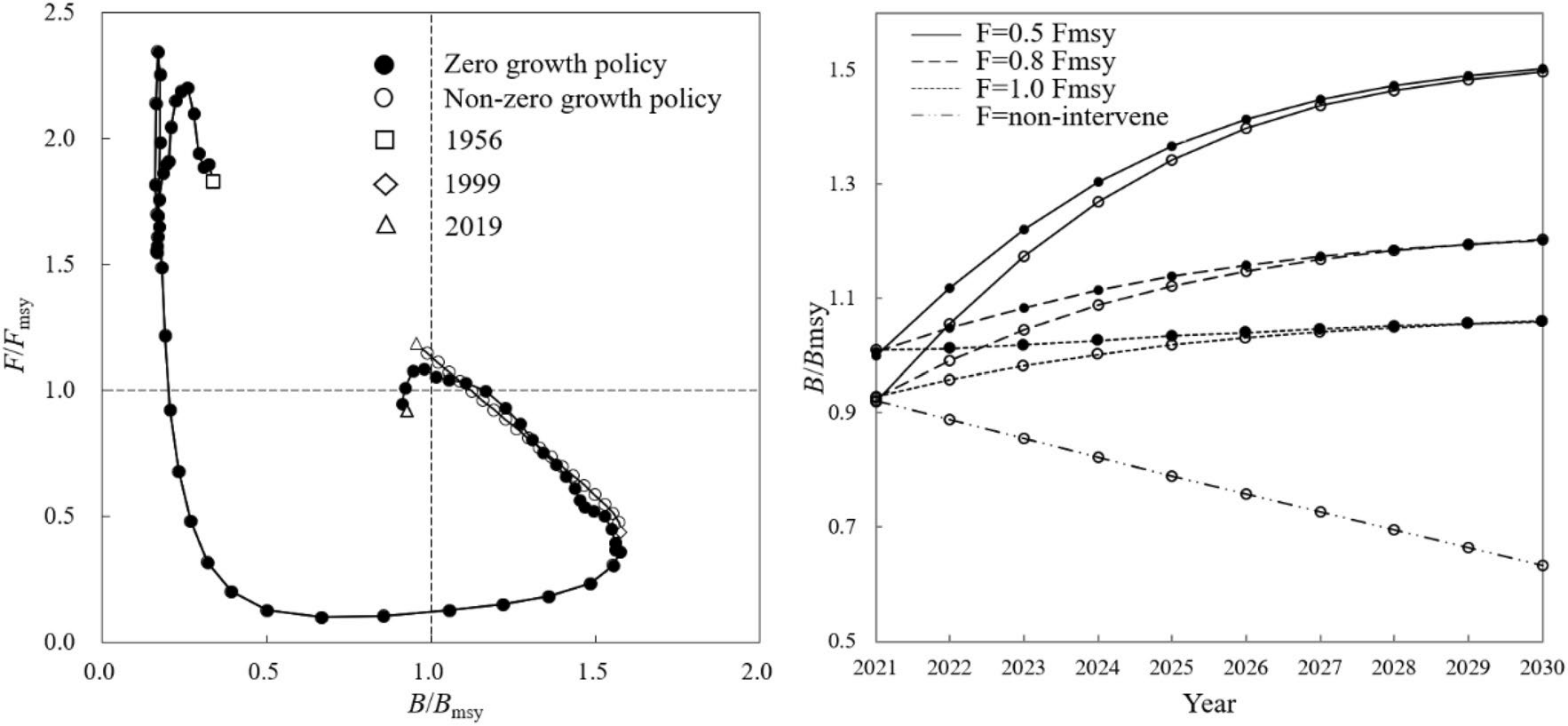
Rebuilding status of *Larimichthys polyactis*. Resource status in different quadrants following explanation in Fig. 2.

*Larimichthys polyactis *experienced a turbulent from overfishing in the 1956–1970s via recovering in the 1980s to a health state in the 1990–2000s, following a decline again. In 2019 *B*/*B*_msy_ was 0.93 and *F*/*F*_msy_ was 0.917, suggesting a beginning of resource depletion (Fig. 7, left). For resource rebuilding, *B*/*B*_msy_ reached 1.12 in 2022 and 1.50 in 2030 under 0.5 *F*_msy_, or 1.01 in 2021 and 1.20 in 2030 under 0.8 *F*_msy_, or 1.01 by 2021 and 1.06 by 2030 under 1.0 *F*_msy_ (Fig. 7, right).

Without the “zero-growth” policy, the state dropped in “depletion” in 2018, with *B*/*B*_msy_ of 0.98 and *F*/*F*_msy_ of 1.15 (Fig. 7, left). Without rebuilding, in 2030 *B*/*B*_msy_ would fall to 0.6. If rebuilding, *B*/*B*_msy_ was expected to 1.05 in 2022 and 1.50 in 2030 under 0.5 *F*_msy_ scenario, with the production of 189,000 T and 265,000 T, respectively, and reached over 1 in 2023 in 0.8 *F*_msy_ scenario and in 2024 in 1.0 *F*_msy_ scenario, with the production of 299,000 T and 338,000 T, respectively (Fig. 7, right).

#### Sardinops sagax

**Figure 8:**
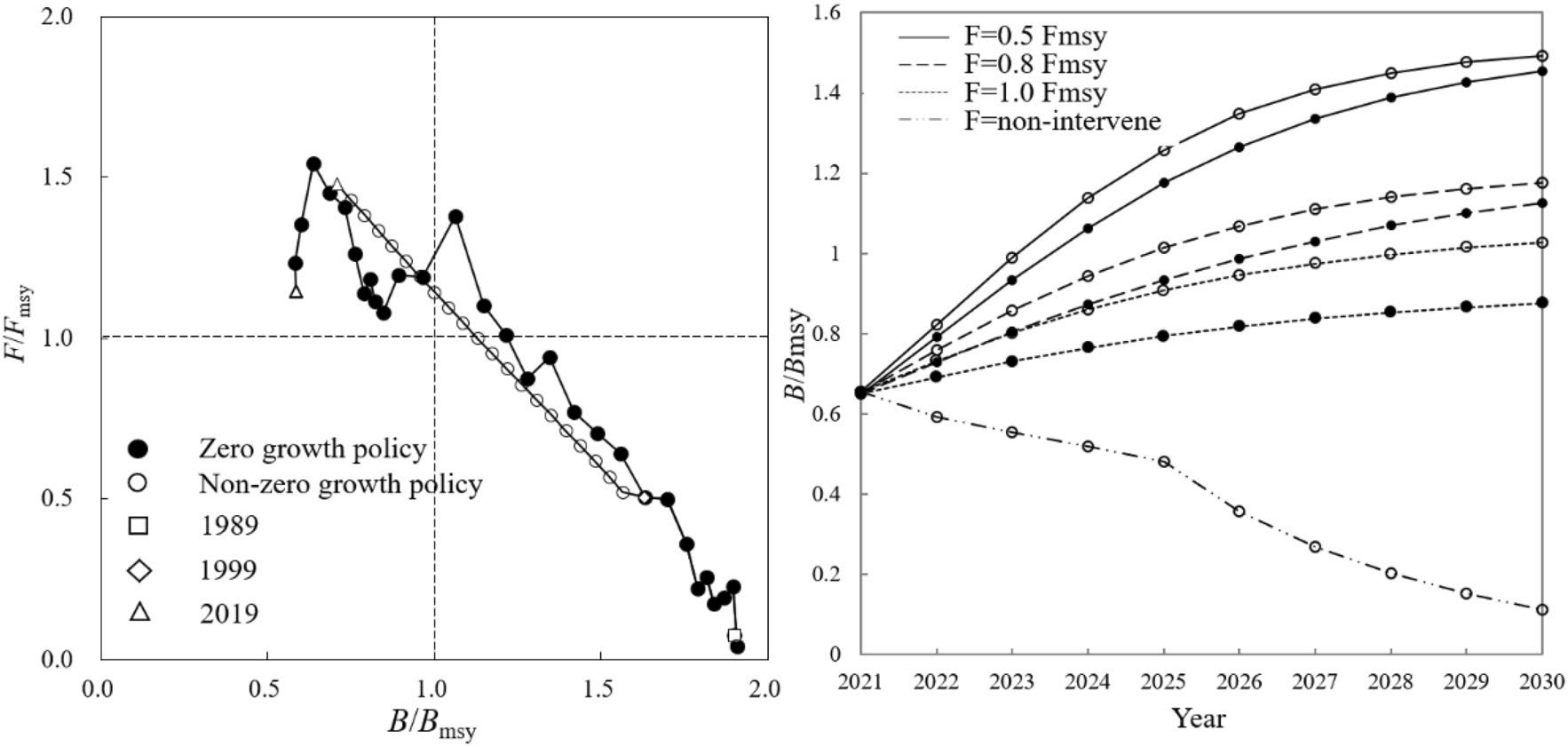
Rebuilding status of *Sardinops sagax*. Resource status in different quadrants following explanation in Fig. 2.

In 1989 the *B*/*B*_msy_ and *F*/*F*_msy_ of *S. sagax* were 1.90 and 0.08, respectively. The resource state fell into “destroying” in 2014 and “depletion” with *B*/*B*_msy_ of 0.97 and *F*/*F*_msy_ of 1.19 (Fig. 8, left). Under different scenarios of resource rebuilding, *B*/*B*_msy_ increased to 1.06 in 2024 and 1.45 in 2030 under 0.5 *F*_msy_, or 1.03 in 2027 and 1.13 in 2030 under 0.8 *F*_msy_, or 0.88 by 2030 under 1.0 *F*_msy_ (Fig. 8, right).

Without the “zero-growth” policy, *F*/*F*_msy_ exceeded 1 for the first time in 2010, and increased to 1.19 when *B*/*B*_msy_ fell below 1 for the first time in 2013, entering the state of “depletion” (Fig. 8, left). Without rebuilding, *B*/*B*_msy_ declined to 0.15 by 2030. If rebuilding, by 2030 *B*/*B*_msy_ could reach 1.40 under 0.5 *F*_msy_ scenario, or 1.18 of 0.8 *F*_msy_ scenario, or 1.03 under 1.0 *F*_msy_ scenario, and the catch could climb to 146,000 T (Fig. 8, right).

#### Portunus trituberculatus

**Figure 9:**
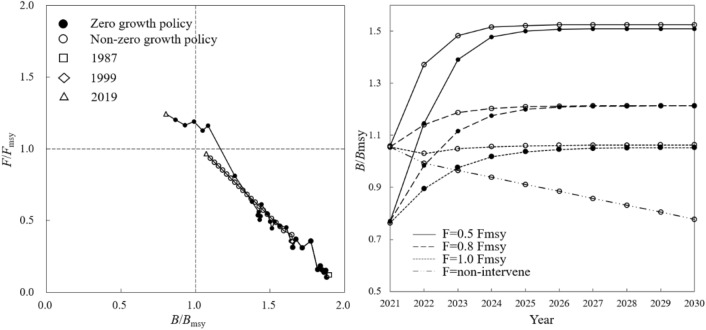
Rebuilding status of *Portunus trituberculatus*. Resource status in different quadrants following explanation in Fig. 2.

Parameters *B*/*B*_msy_ as 1.90 and *F*/*F*_msy_ as 0.12 indicated a “health” state in 1987. The resource yearly declined and fell into “depletion” until 2016 with *B*/*B*_msy_ as 0.99 and *F*/*F*_msy_ of 1.19, and continued to deteriorate to 0.80 of *B*/*B*_msy_ and 1.24 of *F*/*F*_msy_ in 2019 (Fig. 9, left). If rebuilding, *B*/*B*_msy_ increased to a healthy value (> 1) in 2022 under 0.5 *F*_msy_, and delayed to 2023 under 0.8 *F*_msy_. By 2030, an expected catch of 465,000 T at *B*/*B*_msy_ as 1.05 appeared under 1.0 *F*_msy_ (Fig. 9, right).

Without the “zero-growth” policy, though the resource status kept decreasing, it was always in “health” condition until 2019 (*B*/*B*_msy_ as 1.07 and *F*/*F*_msy_ as 0.96), highly likely to enter destruction in the coming year (Fig. 9, left), later than that in practical. For resource rebuilding, the *B/B*_msy_ was expected to 1.52, 1.21 and 1.06 under 0.5 *F*_msy_, 0.8 *F*_msy_ or 1.0 *F*_msy_ by 2030, with the production of 356,000 T, 454,000 T, and 472,000 T respectively. (Fig. 9, right).

#### Cuttlefishes

**Figure 10:**
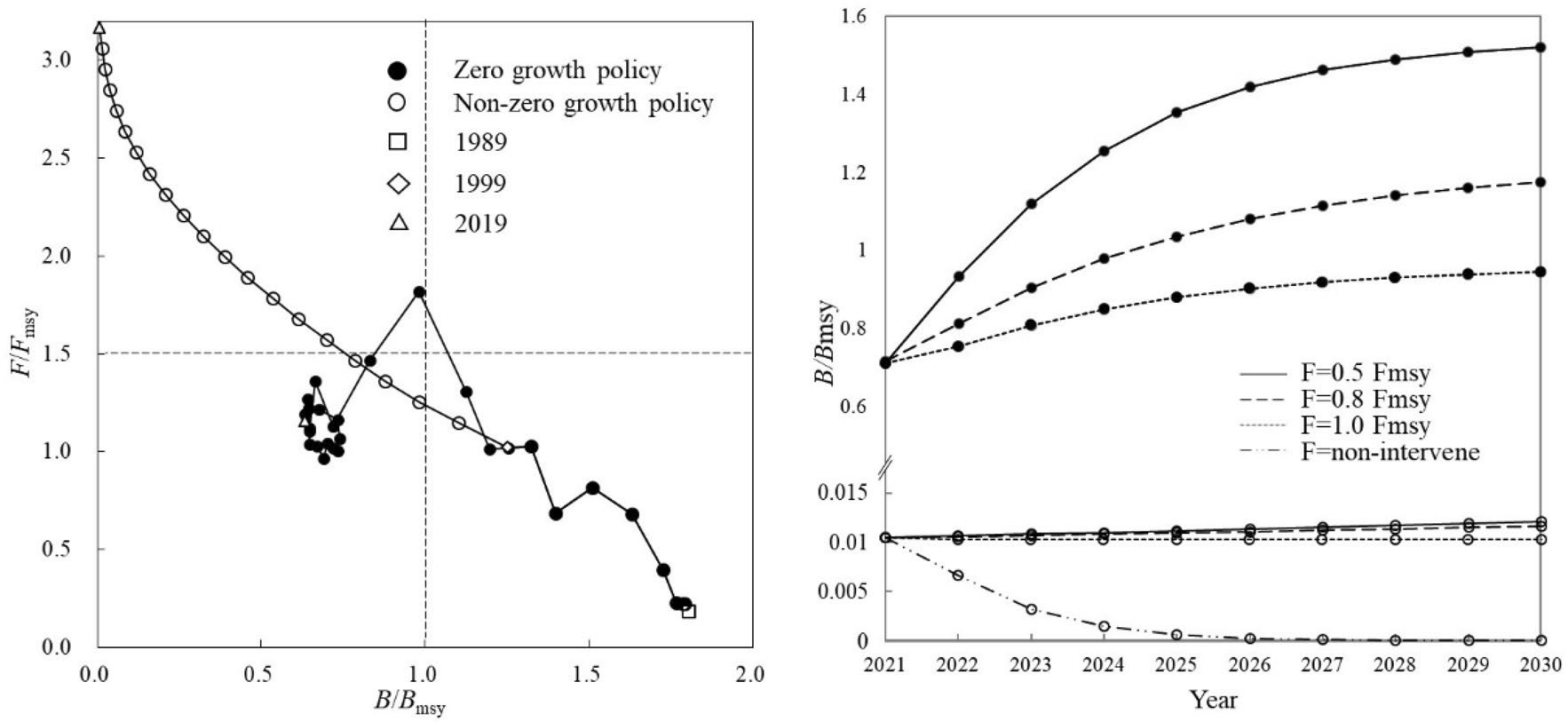
Rebuilding status of the cuttlefishes. Resource status in different quadrants following explanation in Fig. 2.

The state of the cuttlefish resource was “health” in 1989–1996 (*B*/*B*_msy_ > 1 and *F*/*F*_msy_ < 1), “destroying” in 1997–2000 (*B*/*B*_msy_ > 1 and *F*/*F*_msy_ > 1), and later “depletion” (*B*/*B*_msy_ < 1 and *F*/*F*_msy_ > 1) in 2001. In 2019 *B*/*B*_msy_ was 0.63 while *F*/*F*_msy_ was 1.19 (Fig. 10, left). Under 0.5 *F*_msy_, *B*/*B*_msy_ was expected to 1.12 by 2023 and 1.52 by 2030; under 0.8 *F*_msy_, *B*/*B*_msy_ reached a healthy value by 2024; under 1.0 *F*_msy_, even in 2030 *B*/*B*_msy_ was 0.95, lower than 1 (Fig. 10, right).

Without the “zero-growth” policy, the cuttlefish resource showed much more severe tendency of destruction, even to possible collapse in 2019 with *B*/*B*_msy_ close to zero and an extremely high value 3.27 of *F*/*F*_msy_ (Fig. 10, left). Under this simulation, resource rebuild did not work at all at any scenarios (Fig. 10, right).

## Discussion

### Total catch control regulation does not lead to the recovery of fisheries and the maintenance of community function

To contain the decline of wild capture fisheries by overfishing, a series of management regulations have been in place in China to mitigate the fishing impacts as much as possible and maintain sustainable stocks. The “zero-growth” policy is one of the most outstanding representatives. The results showed certain achievements after the implementation of the policy. Simulating the status without the “zero-growth” policy, *B/B*_msy_ fell below 0.5 by 2010 and close to zero by 2019, indicating the impossibility for recovery. However, the policy is not enough for fishery recovery and community health, failing to stop the degradation of fishery resources. Under the implementation of the “zero-growth” policy, *B/B*_msy_ was in a healthy state in 1998, fell below 1 for the first time in 2003, and dropped to 0.52 in 2019, accompanying by *F/F*_msy_ as 1.60. If fishing pressure were maintained at the level of 2019 (*F* = 1.56 *F*_msy_), the resource would decline to the depletion state by 2030 (*B/B*_msy_ close to zero, *F/F*_msy_ = 3.64, catch = 35 T). Therefore, a great degree of negative production growth as well as the strict implementation is extremely important. A rapid reduction in the catch control under 0.5 *F*_msy_ scenario would expect to achieve a quick recovery with *B/B*_msy_ over 1 in 2025. Nevertheless, a significant reduction in production would lead to the decline of fishery economics, livelihood difficulties for fishermen and a series of derivative social problems^28^. An alternative of 1.0 *F*_msy_ would be feasible, under which *B/B*_msy_ could rise to 1 by 2030 with a production of 11.64 MT, close to MSY.

The “zero growth” policy faces some inherent challenges, at least from the point of view of ensuring the sustainable use of individual species stocks. Attention should be also paid at the catch quota control of individual species. Because the variation of the intrinsic growth rate of different species, the *B* is dynamic, and the *F* changes with the change of *B*. In a constant production, *r*-strategic species could remain a higher *B/B*_msy_ than 1 even at a large proportion in catch, but *K*-strategic species did not show the same fortune. The control of total catch volume rather than individual species could not prevent the community structure from becoming fragile, with the exhaustion of high-trophic species and the decrease of mean trophic level.

### Individual species have different responses to overfishing that highly associated with their biological characteristics

#### High trophic level species can be sensitive to overfishing, and difficult to rebuild stocks after collapse

Hairtails *Trichiurus* spp. are the largest contribution group to China marine capture fisheries, at 0.90 MT about 8.3% of the total production in 2020^2^. They are carnivorous and aggressive with a mean trophic level of 4.4, mainly feeding on fishes in the adult stage, and Mysidacea and Euphausiacea in the juvenile stage^29,30^. The spawning seasons of *Trichiurus* spp. are mainly from April to June, and from September to November in Chinese waters^31^.

China coastal areas are excellent foraging and spawning grounds for *Trichiurus* spp, sustaining a large stock size. If the “zero-growth” policy was not implemented since 1999, the resources of *Trichiurus* spp. would be exhausted by 2027, having no possibility to recovery at 1.0 *F*_msy_. Although the total fisheries production has been controlled, and the fishing moratorium period partly covered the spawning seasons of *Trichiurus* spp., their resource continuous declined into a “destroying” state in 2007, due to the time-lag effect of fishing on high trophic level predators characterized by long population doubling time-consuming^32^. Under intensive fishing pressure, *Trichiurus* spp. have showed astonishing fisheries-induced adaption^33^ by reducing the age and size of maturity, which effectively alleviates the decline rate of *B* value, resulting the maintenance of *Trichiurus* spp. capture production. Under the rebuilding scenario of fishing pressure as 1.0 *F*_msy_, *Trichiurus* spp. *B/B*_msy_ rose to 0.87 by 2030, lower than the recovery rate of national total capture fisheries, suggesting the recovery rate of high trophic level species could be slow^34^. Furthermore, in this study fisheries rebuilding only considers the responses of species to fishing pressure, irrespective of a series of factors sensitive to high trophic level species such as pollution and climate change, which indicated a longer period is needed for resource recovery.

#### Middle trophic level species seems non sensitive to total catch control policy

As a representative of middle trophic level species, *L. polyactis* performed different from *Trichiurus* spp. Under high fishing pressure. It forms spawning and over-wintering aggregations between nearshore and offshore waters, as well as vertical migration, rising at dusk and falling at dawn^35^. The spawning season is from mid-February to early May, prior to the national fishing moratorium, indicating young juveniles are in effective protection rather than spawning stock. In the 1950s, *L. polyactis* was one of the few important species in domestic marine capture fisheries in Chinese waters, producing more than 100,000 T annually^5^. The catch volumes then showed a downward trend and fell significantly to less than 50,000 T in the 1960–1980s. After 3 decades low catch volumes, the annual capture production rebounded significantly to more than 200,000 T and maintained at such high levels for 2 decades^5^, showing high resilience to overfishing.

Despite many concerns on the risk of resource exhaustion of *L. polyactis* stocks^5,36^, official statistics showed that the annual catch remains high. The *L. polyactis* production broke through 150,000 tons in 1995, and was above 300,000 tons after 2005. There is likely to have a large offshore stock of *L. polyactis*, which gradually joined the catch under increasing fishing efforts offshore. Furthermore, the *L. polyactis* stocks can be resilience to high pressure for several reasons: (1) its miscellaneous diet makes them be able to receive sufficient food sources; (2) size and age at sexual maturity reduced^37,38^; and (3) the over consumption of top predators relieves the prey pressure on middle trophic level species, such as *L. polyactis*, snappers, and flatfishes. A good job is the difficulties of artificial propagation and seedling breeding of small yellow croaker were broken for the first time in 2015^39^ and the whole artificial cultivation was successfully realized in 2020 (https://www.chinanews.com.cn/cj/2020/07-02/9227715.shtml), which would effectively alleviate the market demand and wild stock sustain of small yellow croaker.

#### Pelagic small fish stocks may not recovery quickly as early cognition

Small pelagic fishes enjoy assembling in large schools of tens of thousands of individuals, and are more vulnerable to predators. Species *S. sagax* mainly filter plankton with a low trophic level about 2.8. It spawns in May–June, with high fecundity (an absolute fecundity of 30,000–100,000 pelagic eggs) and fast growth, and has short generation time of 1.4 years^40^. *S. sagax* shows strong phototropy, and can be caught using light purse seine, gill net, and fixed net fishing at night^41,42^.

In 1989, the biomass of *S. sagax* was about twice of *B*_msy_. With the decreasing capture production of traditional economic fishes, *S. sagax* became a target species using specific fishing methods^43^, resulted in catch increase accompanied with *B/B*_msy_ decline into a state of extremely unhealthy in 2019. Recovery of small pelagic species stocks would be delayed by the total catch control policy, mainly because the removal of large numbers of predator species left more opportunities for their feeding objects^44^. Resource rebuilding of *S. sagax* was not as quick as expected, as small pelagic species had to endure increasing predation pressure from the recovery of high-trophic species under the total catch control. At 1.0 *F*_msy_ scenario, *B/B*_msy_ would be only 0.88 by 2030, in need of a longer period to healthy state.

#### Well-planned restocking can enhance resource recovery

Swimming crab *P. trituberculatus* has high reproductive capacity, with a female can release two to three batches of eggs during a breeding season, and a batch contains about 1–6 million eggs^45^. Under the complementary of existing management measures and restocking programmes, the production of *P. trituberculatus* was kept in a certain amount close to a healthy state, and there is not an urgent need for its stock rebuilding. Since the 1990s, restocking of hatchery-produced larvae of *P. trituberculatus* has been promoted in coastal waters of China. Large-scaled restocking programmes were documented: 33 million larvae were released into the Yellow Sea by Shandong Province in June 2013 (http://hyj.shandong.gov.cn/xwzx/sjdt/201311/t20131120_507389.html); 50.3 million larvae with carapace width over 6 mm were released in the northern Yellow Sea by Liaoning Province in June 2020 (http://nync.ln.gov.cn/fwzx/zxdt/202007/t20200707_3902016.html); 16.1 million larvae were released into the East China Sea by Daishan County of Zhejiang Province in June 2021 (http://www.daishan.gov.cn/art/2021/6/8/art_1383064_59012675.html). What should be of concern is when, where, and how many seedlings are released^46–48^, to maximumly utilize the environmental resources without encroaching on the benefits of other species.

#### Short-living species can be resilience to overfishing

The main cephalopod species in Chinese fisheries are *Sepiella maindroni*, mainly distributes in the East China Sea^35^ and *Sepia esculenta*, mainly distributes in the Bohai Sea, the Yellow Sea and the East China Sea^49^. As a 1-year lifespan species with fast growth rate, *S. maindroni* forms spawning migration from deep water to shallow nearshore bays in spring, partly within the fishing moratorium period. Due to the positive phototaxis, the cuttlefishes can be captured by light seining. *Sepiella esculenta* was the most important cephalopod economically in the northern coastal seas and one of the four major fisheries in the Bohai Sea and the Yellow Sea until the 1970s^50^. The abundance of this species has been greatly reduced with continuous fishing pressures and dwindling spawning grounds^51^.

Total catch control and fishing moratorium showed significant output on the short lifespan cuttlefishes. Without the implementation of the “zero-growth” policy, the cuttlefishes resources would have been exhausted by 2015 and impossible to rebuild. According to the current state of resources, by 2030 the cuttlefish stocks can be recovered under the 1.0 *F*_msy_ scenario. Moreover, the extent of cuttlefishes stock recovery relies on food supply.

### Ways to sustain fisheries

The conflict between rising demand for fishery products and declining resources under multiple pressures including overfishing, climate change, and marine pollution has put heavy pressures at a global scale^52^. Chinese government has undertaken serious reforms to effectively replan the fishery industry.

The effective recovery and rational utilization of resources depend on the support by sufficient reliable data. China started fishery statistics right after the foundation of the People's Republic of China, completed by MOA (1949–2017) and MARA (since 2018). However, the statistical dataset has been questioned internationally^53^. According to the explanation by FAO^54^, before 2000s, especially from 1979 to the late 1990s, as the central government raced to meet the increasing demand for seafood and to grow the domestic production, the local governments had frequently overreported their local catch. In addition, fishermen may falsely claim to increase their production for surplus compensation, after the government introduced fishing subsidies. On the contrary, the production might have been underreported since the early 2000s^55,56^, which could be attributed to the existence of a large number of “black ships” (fishing vessels without relevant legal permits). Moreover, the lack of professionals in the early period and inaccurate knowledge of species identification by fishermen also lead to data uncertainty. Reasonable fisheries data should be consistent with the species functional traits and life history characteristics. However, in the actual fishing activities, the intentional and high-intensity selective fishing of species may greatly deviate the catch data from the data predicted by models. The Chinese government has been trying to improve the statistical system, including data coefficient adjustment, training of fishermen and professional, and supervision of statistical authorities^5^. In this study, selected objects are inshore species: the species are familiar to fishermen; the fishing vessel supervision is in place; the data collection is relatively rational and complete; all these are conducive to the reliability of the results.

The zero-growth policy, which has been implemented since 1999, is an important measure in the history of marine fishery development and management in China. That is, the total catch of marine fisheries in the current year cannot be higher than that of the previous year. However, the “12th Five-year Plan” for national fishery development (2011–2015) issued by the Ministry of Agriculture canceled the mandatory targets of controlling the production but to encourage more catches of marine fisheries (http://www.moa.gov.cn/gk/ghjh_1/201110/t20111017_2357716.htm). In 2013, the State Council published the first state-level marine fishery development document as “Several Advices on Promoting Marine Sustainable and Healthy Development”, incorporating marine fishery development into the strategy of building a maritime power (http://www.gov.cn/zwgk/2013-06/25/content_2433577.htm). This policy shift was clearly reflected in the significant increase in the national annual catch from 12 to 14 MT. Until the “13th Five-year Plan” for national fishery development (2016–2020) issued in 2016, the zero-even negative-growth policy was revalidated, and the volume of annual output control was clearly proposed as 8–10 MT^57^, which was determined by multiplying the fishing coefficient by the total stock size derived from the assessment of surveys on the zoning of fisheries and the supplementary survey of marine biological resources in the exclusive economic zone and the continental shelf^7^. To achieve the target of keeping fishing capacity at a high level of sustainability, significant reductions in fishing pressures over a period of time are required, as well as rational updates of control policies.

Many policies were introduced together or around the same time as the “zero-growth” policy, such as summer fishing moratorium, fishing license system, and fishing fuel subsides. However, the achievements are far from satisfactory. The fishing fuel subsidy policy together with the license system induced the direct fishing vessel construction boom which resulted in fewer but bigger and more powerful fishing vessels. Fishing moratorium is the most promising policy, by leaving enough time and space for fish to successfully reproduce. However, the truth is that, right after the fishing closure season, almost all fishing vessels immediately rush into the sea and fishermen try their best to fish as much as possible within the gears and engine power permission of their fishing licenses, attempting to earn a year's income in a short period of 2–4 months. As a result of such high fishing effort, the achievements of seasonal fishing bans were largely offset and resource densities fell to low levels after autumn. The number of legally binding standards for mesh size is not enough, only 6 at present of at least 40 fishing target species and over 10 fishing gears, leaving many fishing gears and fish species outside the regulation of existing standards^6,58^. Ideally, standards of mesh size should be updated corresponding to the changes of species traits, however, it is a challenge because the main fishing mode is multiple species fishery by bottom trawling. Moreover, species in China seas are diverse, and the spawning period of different species may not fall into the fishing closure season^5^. The lack of specificity to sufficiently cover all the species may result an unbalance of community composition. Another system “Double Control” aims to limit both the numbers of fishing vessels and the total power. Unfortunately, the inspections of fishing vessels and their power are not very strict, due to the need of developing local economy and guaranteeing the fishermen's income, e.g., under a nominal power mask the low-power engines have been replaced by high-power engines, some fishing vessels do not have the fishing licenses^28^. The limitation of the license number and engine power also stimulate the technological improvement for more catch^7^.

The structure adjustment of fisheries composition is the main management measure at present. The high degree of self-sufficiency in fishery products in China has been achieved through overfishing of domestic fishery resources, resulting in the rapid depletion of fisheries in China's coastal waters^59^. Aquaculture, accounting for more than 70% of China's total fisheries production^2^, is identified as a successful way. Accompanying by aquaculture development, a series of problems also arise, particularly, the demand of low-value/trash fish and fish meal that significantly drives further expansion of capture fisheries^60^. Cooperation with other countries to promote regional aquaculture may be an alternative way to meeting global growing demand for seafood and combating overfishing^61,62^. Seeking resources from the high seas and EEZs of other countries is also a choice, of course, on the premise of taking full account of ecology, maritime, and food security of other countries^63–65^.

In addition, this study pointed out a new focus for fisheries management, in which differences in species biological traits, including species vulnerability, population multiplication, and resilience to environmental pressures, should be given full consideration. On this basis, more detailed and targeted management schemes are supposed to propose to achieve the dual purpose of recoverable fisheries resource and balanced species composition, so as to become a truly sustainable fishery. In short, the effective implementation of various management measures is an indispensable guarantee.

## Supplementary Information

Supplementary Information.

## Data Availability

The data underlying this article are available in the online supplementary material.
